# Novel method enabling a rapid vitality determination of cyanobacteria

**DOI:** 10.1002/elsc.201900164

**Published:** 2020-10-27

**Authors:** Marco Witthohn, Anna Schwarz, Jakob Walther, Dorina Strieth, Roland Ulber, Kai Muffler

**Affiliations:** ^1^ Department of Life Sciences and Engineering University of Applied Sciences Bingen Bingen Germany; ^2^ Institute of Bioprocess Engineering University of Kaiserslautern Kaiserslautern Germany

**Keywords:** cell vitality determination, cyanobacteria, *Trichocoleus sociatus*

## Abstract

Cyanobacteria represent a large group of bacteria with underestimated scientific potential. Recent studies indicate them as a great reservoir of secondary metabolites with antifungal, antiviral or antibacterial activity. However, common, well established research techniques cannot be easily adapted to these organisms. Slow growth rates and irregular cell aggregates constitute challenges for researchers dealing with cyanobacteria. In this work, we present an innovative new method enabling a quick, easy and economical vitality determination of cyanobacterial strains, as, e.g. required for the finding of optimal cryopreservation conditions. We were able to measure the vitality of previously cryopreserved and defrosted *Trichocoleus sociatus* samples within 45 min by means of their O_2_‐production. For each run, a cell wet mass of only 0.5 g was required. By application of this method, we could find DMSO (5% v/v) and glycerin (15% v/v) to be the most promising cryoprotectants for the conservation of *T. sociatus* cells. DMSO and glycerin guaranteed a vitality rate of 80–90% and 60–70% after up to four weeks of cryopreservation, compared to fresh cell material.

**Abbreviation**
CDM: cell dry mass

## 1 INTRODUCTION

Cyanobacteria are a group of versatile, phenologically and ecologically diverse microorganisms, which occur in most habitable areas in the world. Because of this circumstance, these organisms are of great interest for scientific research, as they are known as producers of highly valuable secondary metabolites [1, 2, 3], and can be used as phototrophic production strains of high energy compounds, used as biofuels [4]. Although cyanobacteria are not ideal production organisms, mainly due to slow growth rates, new cultivation concepts and reactor types can highly improve the productivity of promising strains [5]. Especially for terrestrial cyanobacteria, the principle of “productive biofilms” is a promising approach [6, 7]. However, a reasonable exploration of these bacteria requires stable and easy to handle strain collections. Cyanobacterial strain collections are commonly sustained as actively growing cell cultures on solid agar plates or in liquid medium [8]. This conservation mode is not only combined with much work effort, but also with high risks of contamination and genetical modifications [9]. Safe conservation methods as freeze drying, the inclusion in alginate beads [10] and, most importantly, cryopreservation [8], have already been adapted for many cyanobacterial strains. Nevertheless, it remains a challenge to define one appropriate method for all strains, due to the high phenological and ecological diversity. For the invention of possible new methods, it is necessary to compare cell vitalities after different approaches of cryoconservation. Regularly used methods are, e.g. the determination of growth through repeated OD measurements, or the colony‐forming unit assay on agar plates. There are also some promising recent approaches, as the staining of cells with a fluorescence colorant, which can only intercalate in the DNA of dead cells [5]. However, these methods are merely realizable for larger laboratories with superior equipment and resources. The classic approaches are though not easily applicable for cyanobacterial strains. Through the formation of cell aggregates or the production of exopolysaccharides, absorption measurement and colony counts often become impractical. As a possible solution on this, a new approach for the determination of cyanobacterial cell viability is presented in this work. The formation of oxygen and the consequently rising pO_2_ value in *T. sociatus* samples was measured by a new method and used for vitality evaluation.

## 2 MATERIALS AND METHODS

### 2.1 Strain cultivation

The cyanobacterial strain *Trichocoleus sociatus* (obtained from Prof. Dr. Burkhard Büdel, University of Kaiserslautern, Department of Plant Ecology and Systematics, Germany) was cultivated in 300 mL flasks, on a rotation shaker at 120 rpm (Multitron Pro, Infors HT, Switzerland) under phototrophic conditions (E_v _= 100 μmol m^−2^ s^−1^) at 27°C, without addition of CO_2_. The standard mineral medium BG‐11 [11] was used for cultivation. The flasks were inoculated with *T. sociatus* cells sustained on solid BG‐11 agar plates under phototrophic conditions (E_v _= 30 μmol m^−2^ s^−1^).

### 2.2 Cryopreservation

Main cultures were inoculated with about 0.25 g wet cell mass from the precultures. Cells were harvested after 4–6 weeks of growth by centrifugation at 2360 x *g* and 25°C for 10 min. (Rotanta 460 R, Hettich, Germany). After cell harvest, the culture supernatant was removed, the cells were carefully mixed with a spatula and cell wet masses of 0.5 g were respectively weighed into cryo‐vials (AEH8.1, Carl Roth, Germany) with a high‐resolution balance. The cell mass was regularly mixed to ensure an equal composition in all tubes. Subsequently, all samples were mixed up to a concentration of 5% v/v with DMSO or MeOH, or up to 15% v/v with glycerin, respectively. All cryoprotectants were diluted with BG‐11 medium. Each sample was done in triplicates. The cryo‐vials were subsequently transferred to a precooled (8°C) passive freezing device (Mister Frosty, Nalgene, USA) and cooled to  −80°C (New Brunswick™ U360 Innova^®^, Eppendorf AG, Germany) with a cooling rate of −1°C min^−1^. Cells were stored at −80°C for up to four weeks. The cells were thawed quickly in a water bath at 30°C for 10 min. Cryoprotectants were removed by three washing steps. Sedimentation was used for cell pelletization instead of centrifugation, because cyanobacterial cells might be sensitive towards centrifugation after cryoconservation [8]. The supernatant was removed and substituted by 1  mL of BG‐11 medium. Afterwards the cryo‐vials were mixed thoroughly. Cell pellets were subsequently transferred to the vitality test setup and mixed up to a volume of 35 mL with BG‐11 medium.

**PRACTICAL APPLICATION**

The presented method is the first approach to link the O_2_ production of cyanobacteria directly to their cell vitality. By this, new conservation methods can be rapidly evaluated, without the need for long cultivation periods, expensive technical equipment or high amounts of biomass. The approach is suitable for every cyanobacterial strain and is not impaired by the commonly occurring production of viscous exopolysaccharides or the formation of cell aggregates. Further application areas are conceivable, for example the testing for antibiotic susceptibility/resistance, or the response to altered cultivation conditions.

### 2.3 Vitality test setup

**FIGURE 1:**
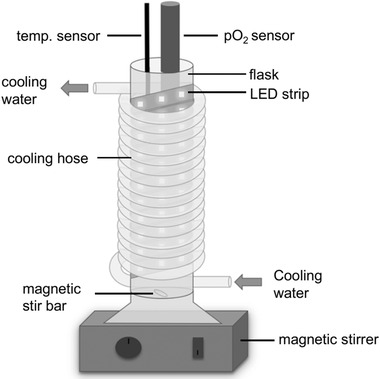
Vitality test setup for cyanobacteria. Cell vitality was determined after cryopreservation by means of pO_2_ increase in the cultivation medium

The verification of cell vitality after cryoconservation was performed with a new measurement technique, based on the pO_2_ alteration by cyanobacterial photosynthesis. The setup (Figure 1) consists of a glass flask, wrapped with an LED strip (Article no. 9180308, Briloner, Germany) for light supply and an overlying cooling hose, connected to a cooling unit (K15, Haake, Germany). The setup is equipped with a temperature sensor (LT‐101, TFA Dostmann, Germany) and a pO_2_ sensor (OxyFerm FDA 225, Hamilton Company, USA). Data of the pO_2_ sensor was collected by a connected bioreactor (Minifors, Infors HT, Switzerland) and monitored with the bioprocess control software IRIS 6 (Infors HT, Switzerland). Medium circulation is assured by a magnetic stirrer (Mini MR Standard, IKA, Germany) at about 500 rpm. Measurements were done for 45 min, at a constant temperature of 27°C. For each run, a cell wet mass of 0.5 g was used.

### 2.4 Determination of cell vitality

Cell vitality of *T. sociatus* was obtained by measuring the pO_2_ and calculation of the highest gradient of the pO_2_ curve. Values obtained from fresh cell material were determined as 100% vital and served as reference value.

### 2.5 Method comparison

The new pO_2_ based vitality test for cyanobacteria was compared to the standard method based on cell mass increase. Therefore, samples of 0.2 g fresh *T. sociatus* biomass were cryopreserved and thawed after two weeks as specified in **2.2**. For each tested cryoprotectant, three 100 mL cultivation flasks were filled with 20 mL BG‐11 medium and inoculated with the respective sample (in triplicates). The cultivation conditions are listed in **2.1**. Flasks were harvested after 3, 7 and 10 days and the cells were washed three times and centrifuged at 3000 x g (EBA 12, Hettich, Germany) with BG‐11 medium. The cell dry mass was determined gravimetrically (M254Ai, BEL engineering, Italy) after drying at 60°C for 24 h.

## 3 RESULTS AND DISCUSSION

### 3.1 Vitality determination via pO_2_ measurement

**FIGURE 2:**
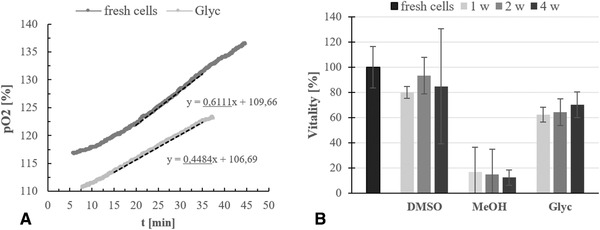
(A) Demonstration of data acquisition through measurement of the pO_2_ increase in the setup. (B), Results of the vitality test with *T. sociatus* cells after cryopreservation with 5% v/v of DMSO, 5% v/v of MeOH or 15% of v/v glycerin as cryoprotectant. The samples were frozen for one, two and, four weeks. The pO_2_ increase in the measurement setup was compared to the one of fresh cells. (Error bars = SD, *n* = 3)

Data concerning the vitality of cyanobacterial cells was gained by measurement of increasing pO_2_ values in the test setup and comparison of the gradients with data from fresh cell material (Figure 2A). By application of the method demonstrated in this work, it was possible to determine significant differences between the three performed cryopreservation approaches, using different cryoprotectants (Figure 2B). While the samples mixed with MeOH showed a clearly diminished vitality of about 0–18 %, the ones cryopreserved with either DMSO or glycerin reached decent activity values of 80–96% and 60–70%. Similar results were obtained with cells of the terrestrial cyanobacterium *Nostoc muscorum*, which were equally cryopreserved and tested for vitality as *T. sociatus* (data not shown). A significant decrease in cell vitality over time was only detectable with DMSO after four weeks of cryopreservation. In case of glycerin as cryoprotectant, the freezing duration had only minimal impact on cell vitality, low standard deviations give hints on consistent amounts of intact cells.

### 3.2 Method validation

**FIGURE 3:**
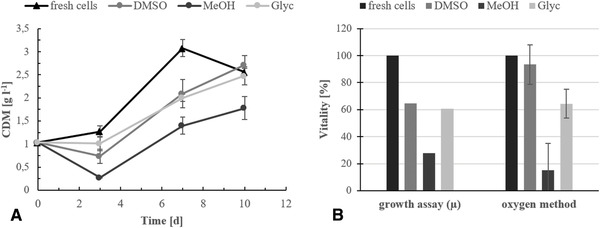
(A) Results of the vitality test (growth assay) with *T. sociatus* cells after two weeks of cryopreservation with 5% v/v of DMSO, 5% v/v of MeOH or 15% of v/v glycerin as cryoprotectant. Data of fresh cells serve as reference. The increase of CDM was measured by cultivation and gravimetrical evaluation of *T. sociatus* samples after different modes of cryopreservation. The cells were harvested after 3, 7, and 10 days. For the t_0_ measurement, 200 mg of fresh *T. sociatus* cells were dried at 60°C for 24 h and the CDM was determined gravimetrically. (B) Approximate growth rates μ [h^−1^]; CDM values of t_0_ and t_7_ were considered for calculation. (Error bars = SD, *n* = 3)

The developed technique was validated by comparison with the known standard method, the evaluation of growth experiments (Figure 3). For this, the increase of *T*. *sociatus* cell dry weight was measured gravimetrically after different points of cultivation time (**2.5**). Prior to the vitality assay, the cells were cryopreserved under addition of DMSO, MeOH or glycerin, as done with the cells applied in the pO_2_ measurements (**2.2**). This method was chosen because optical density measurements are impractical, due to inhomogeneous cell aggregates of *T. sociatus*. Cultures inoculated with 10 g L^−1^ *T. sociatus* cell wet mass (1 g L^−1^ cell dry mass, CDM) were harvested after 3, 7 and 10 days of phototrophic cultivation (**2.1**).

The results obtained from the growth assay could confirm the data from the pO_2_ based vitality measurements. The obvious loss of CDM in the growth assay with cryopreserved samples indicates a diminished number of vital cells, due to the freezing process. Cells cryoconserved with MeOH showed the highest difference in CDM between t_0_ and t_3_. Cells frozen with glycerin seem slightly less damaged by the process, in comparison to DMSO samples. The higher CDM value after three days of cultivation can be explained by glycerin residues in the samples, which remained after the washing steps. *T. sociatus* was found to be capable of mixotrophic growth with different carbon sources, resulting in significantly higher growth rates (Schwarz et al., unpublished data). As shown in Figure 3A, the cell vitality determined by pO_2_ measurement allows a relatively precise inference to the corresponding growth rates and thus permit a quick prediction on cell growth behavior. The comparability of both data sets might be improved by more repetitions in the pO_2_ measurements and more CDM values at different points of the growth curve.

## 4 CONCLUDING REMARKS

Considering all obtained results, a simple yet effective novel method for the vitality determination of cyanobacterial cells could be established. The test can approximately predict the growth impairment caused by cryoprotective agents and can give a first hint on negative impacts, which is sufficient for a fast selection of suitable cryoprotectants. The functionality and validity of the assay was confirmed by ‘classic’ growth experiments after identical cell treatment. At this point it should be noted that one growth assay took ten days and 2.4 g of wet cell mass, while the vitality determination by pO_2_ measurements provided results after about 30 min and only required a maximum of 1.5 g wet cell mass. The applied cell mass can also be significantly reduced, when more time is invested in data acquisition. This makes the method feasible for cyanobacteria with very low growth rates, without the need for long cultivation times. Moreover, the method offers great potential for further application fields, as the testing for antibiotic susceptibilities, or reaction of cyanobacterial cells to different medium supplements.

## CONFLICT OF INTEREST

The authors have declared no conflict of interest.
